# A high-quality Oxford Nanopore assembly of the hourglass dolphin (*Lagenorhynchus cruciger*) genome

**DOI:** 10.1093/g3journal/jkaf044

**Published:** 2025-02-28

**Authors:** Nick McGrath, Jamie le Roux, Annabel Whibley, Alana Alexander, Ramari Oliphant Stewart, Muriel Johnstone, Karen A Stockin, Olin K Silander

**Affiliations:** School of Natural Sciences, Massey University, Albany, Auckland 0623, New Zealand; School of Natural Sciences, Massey University, Albany, Auckland 0623, New Zealand; Grapevine Improvement, Bragato Research Institute, Engineering Drive, Lincoln 7647, New Zealand; School of Biomedical Sciences, University of Otago, 290 Great King Street, Central Dunedin, Dunedin 9016, New Zealand; Department of Anatomy, University of Otago, 270 Great King Street, Central Dunedin, Dunedin 9016, New Zealand; Te Kauika Tangaroa Charitable Trust, P.O. Box 110, Franz Josef Glacier, Westland 7856, New Zealand; Ōraka-Aparima Rūnaka, 175 Palmerston Street, Riverton/Aparima 9822, New Zealand; School of Natural Sciences, Massey University, Albany, Auckland 0623, New Zealand; School of Natural Sciences, Massey University, Albany, Auckland 0623, New Zealand; The Liggins Institute, University of Auckland, 85 Park Road, Grafton, Auckland 1023, New Zealand

**Keywords:** Oxford Nanopore, hourglass dolphin, *Lagenorhynchus cruciger*, *Sagmatias*, taonga

## Abstract

The hourglass dolphin (*Lagenorhynchus cruciger*) is a small cetacean species of the Southern Ocean, with significance to iwi Māori (*Māori tribes*) of Aotearoa New Zealand as taonga (*treasured/valued*). Due to the remoteness and difficulty of surveying Antarctic waters, it remains one of the least-studied dolphin species. A recent stranding of an hourglass dolphin represented a rare opportunity to generate a genome assembly as a resource for future study into the conservation and evolutionary biology of this species. In this study, we present a high-quality genome assembly of an hourglass dolphin individual using a single sequencing platform, Oxford Nanopore Technologies, coupled with computationally efficient assembly methods. Our assembly strategy yielded a genome of high contiguity (N50 of 8.07 Mbp) and quality (98.3% BUSCO completeness). Compared to other Delphinoidea reference genomes, this assembly has fewer missing BUSCOs than any except *Orcinus orca*, more single-copy complete BUSCOs than any except *Phocoena sinus*, and 20% fewer duplicated BUSCOs than the average Delphinoidea reference genome. This suggests that it is one of the most complete and accurate marine mammal genomes to date. This study showcases the feasibility of a cost-effective mammalian genome assembly method, allowing for genomic data generation outside the traditional confines of academia and/or resource-rich genome assembly hubs, and facilitating the ability to uphold Indigenous data sovereignty. In the future, the genome assembly presented here will allow valuable insights into the past population size changes, adaptation, vulnerability to future climate change of the hourglass dolphin and related species.

## Teaser

The hourglass dolphin is a taonga (treasured) species to the indigenous peoples (Māori) of New Zealand. Here, in collaboration with Māori researchers, the authors use Oxford Nanopore sequencing to assemble a high-quality genome. This assembly is one of the most complete of any cetacean. The sole use of Oxford Nanopore sequencing highlights that economical and accurate assembly of large genomes is possible. The data from this study is archived on the Aotearoa Genomic Data Repository to uphold data sovereignty of the Indigenous group enabling this research, Ōraka-Aparima.

## Introduction

The hourglass dolphin (*Lagenorhynchus cruciger*) is a small cetacean species that inhabits pelagic Antarctic and sub-Antarctic waters. Although its northernmost range includes Aotearoa New Zealand, where it is considered a taonga (*treasured*) species by iwi Māori (*Māori tribes*), the hourglass dolphin is strongly associated with the Antarctic Convergence, and rarely found in close proximity to land masses (Dellabianca *et al*. 2012; Santora 2012; Acevedo *et al*. 2017). Although not an uncommon species (Goodall *et al*. 1997), due to the remoteness and difficulty of surveying Antarctic waters, it is one of the least-studied species of dolphin (Goodall *et al*. 1997; Dellabianca *et al*. 2012), with information largely limited to observations from living animals: group size, locality, acoustics, and presence of calves (Goodall 1997; Thiele *et al*. 2000; Kyhn *et al*. 2009; Tougaard and Kyhn 2009; Dellabianca *et al*. 2012; Acevedo *et al*. 2017; Todd and Williamson 2022).

For an enigmatic oceanic species such as the hourglass, the few beach stranding events that have occurred have provided important opportunities to learn more about the physiology and other phenotypes such as diet preference (e.g. Goodall *et al*. 1997; Fernandez *et al*. 2003; Marchesi *et al*. 2016; Peters *et al*. 2022). Physical specimens from a stranding also provide an opportunity to obtain genetic material for genome sequencing. These genomes can be a pathway toward understanding past population size changes, adaptation, and vulnerability to changing climate—an aspect particularly important for the hourglass dolphin in the rapidly changing Antarctic environment (MacLeod 2009).

In addition, considerable uncertainty remains around the evolutionary relationships between Lissodelphininae—the subfamily of true dolphins to which the hourglass dolphin belongs (Cope 1866; Goodall *et al*. 1997; Leduc *et al*. 1999; Harlin-Cognato and Honeycutt 2006; McGowen 2011; Banguera-Hinestroza *et al*. 2014; Vollmer *et al*. 2019). Recent analyses combining molecular and morphological data suggest that the *Lagenorhynchus* genus is not monophyletic and that *Lagenorhynchus* should be maintained only for the white-beaked dolphin (*L. albirostris*). Consequently, the hourglass dolphin (*L. cruciger*), the Pacific white-sided dolphin (*L. obliquidens*), and the dusky dolphin (*L. obscurus*) would be assigned to the genus *Sagmatias*, allied with Peale's dolphin, *Sagmatias amblodon* (Cope 1866; Leduc *et al*. 1999; Vollmer *et al*. 2019). Genomic data could be particularly useful in resolving such taxonomic confusion, as demonstrated recently in the taxonomic placement of the pygmy right whale (*Caperea marginata*) (Dutoit *et al*. 2023).

However, the generation and analysis of genomic data can exacerbate pre-existing inequities around who has access to the technology to generate such genetic resources, who gets to decide how these resources are looked after, and who benefits from the generation of such resources (Mc Cartney *et al*. 2022; Te Aika *et al*. 2023). This situation is particularly heightened when it comes to sequencing species that are taonga (*treasured*) by Indigenous peoples. In these cases, the tikanga (*protocols*) around protecting and ensuring the safety of tissues and genetic resources is paramount for upholding Indigenous data sovereignty (Jennings *et al*. 2023; Robbins *et al*. 2023).

One pathway toward upholding Indigenous data sovereignty, and ensuring that capacity-building can occur within the communities where rare species are found, is to develop methods for sequencing and genome assembly that are achievable locally. A primary method for doing so is sequencing using the Oxford Nanopore platform. However, a recurring issue has been that such assemblies are often not of high quality, with low completeness and contiguity (Hauff *et al*. 2025).

Here, we provide evidence that highly accurate assembly is possible for mammalian-sized genomes solely relying on sequence from the Oxford Nanopore Technologies sequencing platform. We compare 2 basecallers (Guppy and Dorado) and 3 assemblers (Raven, Nextdenovo, and Goldrush), obtaining the most contiguous genome using the Dorado basecaller and the computationally efficient Raven assembler. For most mammalian species, obtaining data such as that utilized in our study is achievable on a single Oxford Nanopore PromethION flow cell for ∼$1,500. With the use of a low-overhead RAM assembler such as Raven, it is possible to implement the complete assembly pipeline on a high-end gaming laptop. Finally, we show that this assembly is, on average, more complete than any other published Delphinoidea genome (fewer missing BUSCOs and more complete single-copy BUSCOs), including the highly curated bottlenose dolphin (*Tursiops truncatus*).

While reduced capital outlay and sequencing costs have already allowed for a more equitable distribution of the capability to generate genomic resources, this has rarely extended to include organisms with large or complex genomes. The results here suggest that current genomic technologies can be applied to enhance the ability of Indigenous communities to maintain sovereignty over tissues and data collected from their taonga (*treasured*) species, including rarely sampled species such as the hourglass dolphin.

## Methods and materials

### Specimen collection

On the of August 5, 2020, an individual hourglass dolphin was reported beached at Orepuki Beach in Te Waewae Bay, Murihiku, Aotearoa New Zealand. This individual was given the customary name Hārua-tai, reflecting its connection to the rough seas of the Southern Ocean. With the permission and full blessing of the tribal authority, Ōraka-Aparima Rūnaka, we retrieved and necropsied the dolphin on the 29th of September to recover a full suite of samples from major organs including the heart. The customary name, Hārua-tai, was used in addition to a scientific coding system in order to link the whakapapa (*connections*) of the materials recovered from this animal. Cardiac tissue was stored at −80°C until permission from Ōraka-Aparima was granted to undertake this codesigned study to assemble the genome of the hourglass dolphin. In particular, we aimed to utilize technologies that allowed the library preparation, sequencing, assembly, and archiving of the resulting assembly to remain within New Zealand, consistent with protection of taonga (*treasured possessions*) under Te Tiriti o Waitangi (the Treaty of Waitangi), and to ensure analyses and presentation of findings was conducted in consultation with Ōraka-Aparima.

### DNA isolation and sequencing

We divided the cardiac tissue into 2 subsamples (21 and 25 mg) and isolated DNA from each using the Monarch Genomic DNA Purification Kit. We lysed the tissue for a total of 6.5 h, agitating at 600 rpm for the first 30 min and 300 rpm thereafter. During both these lysis-agitation steps, we incubated the sample at 56°C. We prepped each DNA sample for Oxford Nanopore sequencing using the ligation sequencing kit (SQK-LSK114) according to the manufacturer's instructions. We sequenced the samples on 2 sequential days on 2 PromethION R10.4.1 flow cells on a P2 Solo instrument for 68.5 and 72 h. We stored the DNA at −20C for 3 months and performed a third sequencing run using the latter sample, which had exhibited a higher read N50 (7.2 kilobase pairs (kbp) vs 9.5 kbp).

### Bioinformatics pipeline overview

**Fig. 1.**
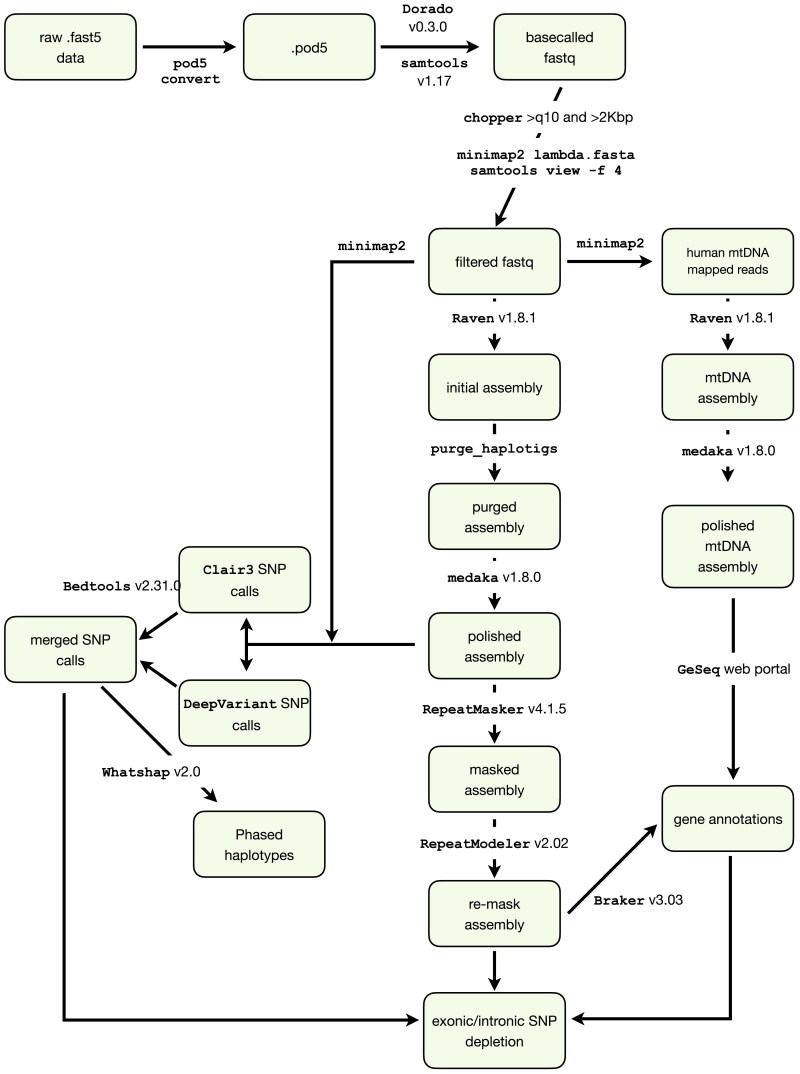
Flowchart of Raven-based assembly. Each software program is noted in Courier serif font; boxes show the results of each step; see methods for details on versions and specific arguments.

We implemented a straightforward strategy for basecalling and filtering, assembly, assessment of assembly contiguity and completeness, haplotig purging, polishing, annotation, and variant calling and phasing (Fig. 1).

### Basecalling and filtering

We basecalled the fast5 files using *Guppy* v.6.3.9. with the dna_r10.4.1_e8.2_400bps_sup model. We also converted fast5 files to pod5 files using *pod5 convert* from the pod5 package of tools. We basecalled the pod5 files using *dorado* v0.3.0 (dorado 2023) and the dna_r10.4.1_e8.2_400bps_sup@v4.1.0 model and converted the resultant.bam file to.fastq format using *samtools fastq* v1.17 (Danecek *et al*. 2021). To remove control DNA, we first mapped all reads to the control lambda phage genome using *minimap2* (Li 2018) and removed all mapped reads using *samtools view -f 4*. We filtered the fastq files using *chopper* (De Coster and Rademakers 2023) to retain only reads with average quality scores above 10 and lengths >2 kbp. We also cropped 50 bp from the head and tail of each read. Using 16 threads, this process took 20 min and minimal RAM. For downsampling the read data to 90 Gb, we used filtlong (Wick and Menzel 2017), prioritizing read length (−length_weight 10).

### Assembly

We then assembled both the Dorado-called and Guppy-called data using 3 different assemblers with RAM requirements <400 GB, and which are optimized for Oxford Nanopore data: Goldrush v1.0.1 (Wong *et al*. 2023), Nextdenovo v2.5.2 (Hu *et al*. 2024), and Raven v1.8.1 (Vaser and Šikić 2021). We selected these assemblers due to their active maintenance, established performance with long reads, and relatively low compute requirements. For example, using Raven with the Dorado data took a total time of 16 h and a maximum of 153 Gb RAM, averaging ∼130 Gb of RAM (Supplementary Fig. 1 in File 1). Our available compute resources precluded the use of some other assemblers such as Flye (Kolmogorov *et al*. 2019), which can consume up to 1 Tb of RAM for mammalian-sized genomes.

For mitochondrial genome assembly, we mapped all reads to the human mtDNA sequence, filtered those reads using chopper (De Coster and Rademakers 2023) to include only reads between 14kbp and 17 kbp (the approximate size of the full length mitochondrial genome), and subsampled this filtered set using *SeqKit* (Shen *et al*. 2016) to include only 5% of the reads (due to the extremely high coverage). We assembled these reads using Raven.

We calculated NG50 using Quast v5.2.0 (Gurevich *et al*. 2013), assuming a genome size of 2.384 Gbp.

### Haplotig purging and assembly polishing

We used purge_haplotigs (Roach *et al*. 2018) to remove contigs from the assembly that had lower than expected coverage or which were highly similar to other contigs; these were judged as being haplotigs.

We used medaka v1.8.0 (medaka 2023) to polish the assembly using the *r1041_e82_400bps_sup_v4.1.0* model. Previous work (Flack *et al*. 2023) suggests that medaka alone performs better than medaka followed by NextPolish (Hu *et al*. 2020), or Racon (Vaser *et al*. 2017) alone.

In the most contiguous assembly, Dorado-basecalled data assembled with Raven, we found a short contig of 1,396 bp, with the next smallest contig at almost 25 kbp. Upon blasting this sequence, we found it was 99% identical to a sequence from *Bos taurus*. We removed this contig from the assembly, a likely contaminant from other lab samples. We also used Kraken2 (Wood *et al*. 2019) to determine the taxon assignment for all other contigs in the assembly and found that all were vertebrate in origin, suggesting no microbial contamination. In addition, given the above contaminant *B. taurus* contig, we used mash (Ondov *et al*. 2016) to calculate approximate distances for each contig to the reference *B. taurus* genome and the *T. truncatus* genome using 500,000 min-hashes. All contigs were closer to *T. truncatus* than *B. taurus*, although 84 contigs were too small to accurately measure distance using mash. In these cases, we mapped the contigs to each reference genome using minimap2-x lr:hq (Li 2018). For each contig, we found the longest aligned region to either genome and tested whether the *B. taurus* or the *T. truncatus* alignment was longer. The *T. truncatus* alignments were longer except for 3 contigs with short aligned regions. To test the identity of these contigs, we blasted them against the nr database. The 3 contigs ranged from 93 to 97% identity to other Delphinid genomes. Thus, we concluded that no other contigs besides the short 1,396 bp above were contaminants.

To map the correspondence of the *L. cruciger* hourglass assembly contigs to the autosome and sex chromosomes of the *T. truncatus* assembly, we aligned the *L. cruciger* assembly to the *T. truncatus* assembly using quarTeT Assembly Mapper (Lin *et al*. 2023).

### Repeat regions

We masked repeats using RepeatMasker v4.1.5 (Bogdahn 2015) with the Dfam v3.7 repeat element database, nhmmscan version 3.3.2, and taxa search limited to mammals. After masking these from the genome, we used RepeatModeler v2.02 to identify repeats de novo. Finally, using this library of de novo elements, we used RepeatMasker to search for and subsequently mask any remaining elements in this premasked genome.

We identified telomeric repeats (TTAGGG) using SeqKit (Shen *et al*. 2016). To calculate kmer repetition in contigs, we used jellyfish 2.2.10 (Marçais and Kingsford 2011). For each contig, we counted the frequency of all 21-mers, and then calculated the fraction of 21-mers that were present once vs more than once. We used SeqKit (Shen *et al*. 2016) to count N content across regions masked by RepeatMasker.

### Assembly completeness

We assessed assembly completeness using compleasm.py (Huang and Li 2023) and the odb10 versions of 5 databases: Laurasiatheria, Cetartiodactyla, Eutheria, Mammalia, and Eukarya. We compared the assembly here to 9 additional Delphinoidea assemblies (Supplementary Table 1 in File 1) to which we applied compleasm in the same way.

### Annotation

We annotated the masked genome using Braker v.3.0.3 (Lomsadze *et al*. 2005; Stanke *et al*. 2006, 2008; Gotoh 2008; Iwata and Gotoh 2012; Buchfink *et al*. 2015; Hoff *et al*. 2016, 2019; Brůna *et al*. 2020, 2021) and the OrthoDB11 Vertebrate database of protein families (March 3, 2023) (Kuznetsov *et al*. 2023) downloaded from https://bioinf.uni-greifswald.de/bioinf/partitioned_odb11/.

We annotated the mitochondrial genome using GeSeq (Tillich *et al*. 2017) with the BLAT reference sequences set to all Delphinid mtDNA genomes in RefSeq.

### Variant calling and phasing

To call variants, we mapped the reads to the purged and polished assembly. We used Clair3 v1.0.4 (Zheng *et al*. 2022) to call variants using the Clair3 *r1041_e82_400bps_sup_v410* model. We observed a clear dip in the number of SNV calls at a quality score of 14 (Supplementary Fig. 3 in File 1), and using *rtg vcffilter* v3.12.1 (Cleary *et al*. 2014), we filtered the calls to include only those with qualities above 14. We also used DeepVariant (Poplin *et al*. 2018) with *–model_type = ONT_R104* and no additional organism-specific training. Both DeepVariant and Clair3 have been shown to perform well with Oxford Nanopore data for SNP and indel detection in bacteria (Hall *et al*. 2024), and Clair3 alone performs well for human variant calling (Nyaga *et al*. 2024). For DeepVariant, there was a clear dip in the number of calls at a quality score of 20 (Supplementary Fig. 2 in File 1), and we retained only calls with scores above that. Finally, we obtained a set of high-quality calls by intersecting these 2 call sets using *bcftools* v1.17 (Danecek *et al*. 2021) *norm* and *isec*. We then used bedtools v2.31.0 (Quinlan and Hall 2010) to find intersections between individual bed files of genic, exonic, and intronic regions and these variant calls.

We phased variant calls using *Whatshap* v2.0 (Martin *et al*. 2016). To visualize the phasing, we used *whatshap haplotag* and IGV v2.1.62 (Thorvaldsdóttir *et al*. 2013).

## Results and discussion

Using Dorado, overall we obtained 8.51 million reads and 39.6 Gbp; 4.96 million reads and 31.3 Gbp; and 20.4 million reads and 110.2 Gbp of data from the 3 Oxford Nanopore runs, respectively. The N50 values for these runs were 7.2, 9.5, and 8.4 kbp. After pooling these 3 runs and filtering, using Dorado, 20.3 million reads and 142.6 Gbp remained, with an N50 of 9.04 kbp; using Guppy and filtering (length >2 kbp and quality >10) resulted in 21.1 million reads and 145.9 Gbp, with an N50 of 8.88 kbp. Using both datasets and all 3 assemblers (Goldrush, Raven, and Nextdenovo), we found that contiguity differed considerably (Table 1), with the Dorado-basecalled Raven assembly having the longest contig NG50 and maximum contig length (8.08 and 39.1 Mbp, respectively. Both of these are almost 50% longer than the least contiguous method, Guppy with Goldrush.

**Table 1.** Assembly contiguity across basecallers and assemblers.

Assembler	Basecaller	Number of contigs	Total length (Gbp)	Min. contig length	Max. contig length (Mbp)	NG50 (Mbp)
Goldrush	Guppy	40,014	2.57	1	28.7	5.68
Goldrush	Dorado	29,863	2.44	1	24.5	6.29
Nextdenovo	Guppy	1,090	2.32	15,220	28.4	6.55
Nextdenovo	Dorado	1,098	2.32	16,390	27.5	6.42
Raven	Guppy	1,289	2.45	1,380	24.9	7.32
Raven	Dorado	1,243	2.44	1,379	39.1	8.08
Raven	Dorado Q16	1,187	2.40	10,313	30.6	6.38

The statistics below are for assemblies before polishing, haplotig purging, or contaminant removal.

We obtained 31 Mbp of reads (subsampled to include only 5% of the total number) between 14 and 17 kbp that mapped to the human mitogenome, with a mean length of 15,923 bp. Using Dorado-basecalled data and Raven (the most successful whole-genome combination), we assembled a circular 16,389 bp mitochondrial contig. This was 96.9% identical to the 16,392 bp Pacific white-sided dolphin *L. obliquidens* mitogenome (Lee *et al*. 2018) and 96.6% identical to the 16,371 bp Heaviside's dolphin *C. heavisidii* mitogenome (Hassanin *et al*. 2012), the 2 closest matches in the NCBI database.

### Assembly completeness across assemblers

We quantified assembly completeness using compleasm (Huang and Li 2023 Sep 27), a new implementation of BUSCO (Simão *et al*. 2015) using the Laurasiatheria odb10 database. Again, we found that the Dorado-basecalled Raven assembly had the highest number of complete single-copy BUSCOs and the fewest fragmented or missing BUSCOs compared to the other 5 basecaller-assembly combinations (Supplementary Table 2 in File 1). Due to the Dorado-based Raven assembly having the highest contiguity and BUSCO completeness, we used this assembly as the basis for the remainder of the analyses here.

### Haplotig purging and assembly polishing

We performed a single round of haplotig purging and polishing, resulting in a final assembly 2.384 Gbp in length, with 894 contigs, an NG50 of 8.074 Mbp, an L50 of 89, a maximum contig length of 39.03 Mbp, and a minimum contig length of 24,985 bp (Supplementary Fig. 2 in File 1). This assembly size is similar to the total chromosomally scaffolded portions of other delphinid species such as the bottlenose dolphin *T. truncatus* (2.343 Gbp), the common dolphin *Delphis delphinus* (2.364 Gbp), the white-beaked dolphin *Lagenorhynchus albirostris* (2.404 Gbp) and the long-finned pilot whale *Globicephala melas* (2.364 Gbp). However, it is considerably smaller when including the unplaced scaffolds of these assemblies (2.637, 2.774, 2.767, and 2.651 Gbp, respectively). This may be due to the small repetitive contigs in this *L. cruciger* assembly being artifactually collapsed.

**Fig. 2.**
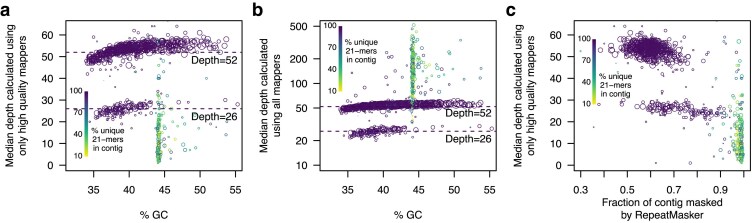
Sequencing depth, GC-content, and repetitiveness varies across contigs. a) GC-content vs depth calculated using high-quality mappers. Each contig is represented by a circle that is scaled to the log of contig size. Depth was largely bimodal, with the lower mode (∼26) corresponding to the X and Y chromosomes in this male individual. A slight bias toward lower depth in very GC-poor contigs was apparent (visible as an upward slope as % GC increases). A small number of contigs exhibited very similar %GC values (∼44%); these contigs had low nucleotide diversity as measured by kmer diversity (green and yellow circles). The low nucleotide diversity of these contigs also resulted in very few high-quality mappers and thus low depth. b) GC-content vs depth calculated using all mapped reads. The same plot as in (a) but when all mapped reads are included. In this case, contigs with low nucleotide diversity exhibited extremely high depth, suggesting that most arose from hard-to-resolve genomic regions that were collapsed during assembly. Note that the scale of the y-axis is linear for (a) and logarithmic for (b) due to the high coverage values for some repetitive contigs (up to 500-fold). c) Masked contig fraction vs depth. Colors are identical to (a) and (b). The x-axis indicates the fraction of each contig that was designated by RepeatMasker as containing repetitive elements (e.g. LINEs, SINEs, and LTRs) and thus masked with Ns. Contigs that have low nucleotide diversity consist almost exclusively of de novo*-*identified repetitive elements, being masked only after annotation of lineage specific repetitive elements.

To estimate the copy number of each contig, we mapped the raw reads back onto the assembly and filtered the mapped reads to include only those with a mapping quality >20. We found that nonrepetitive contigs (see below) had largely bimodal read depths, with the majority having a depth of ∼52 and a smaller number having a depth of ∼26 (Fig. 2), which we inferred were contigs belonging to the X and Y chromosomes in this male individual. We confirmed this by aligning the contigs to the *T. truncatus* genome assembly (a female individual). We found that out of the 129 nonrepetitive contigs with median depth <30, 112 aligned to the X chromosome, 4 did not align, ten aligned to unplaced scaffolds, and 3 aligned to autosomes.

The *T. truncatus* is a female individual and thus has no Y chromosome. Of the 4 unaligned contigs (176, 103, 74, and 70 kbp in length), the top blast hits by bit score were, respectively: *B. taurus* chr Y (80% identity across the alignment, but 93% identity to *D. delphis* chr 12); *G. melas* chr 19 (95% identity); *Balaenoptera acutorostrata* chr 19 (81% identity), and *D. delphis* chr Y (92% identity). Thus, it appears that some unmapped contigs in this hourglass assembly are homologous to other species’ Y chromosomes. The match of a single contig with both the *B. taurus* Y chromosome and *D. delphis* chr 12 suggests that other assemblies may have sex chromosome contigs assigned to autosomal contigs.

A number of contigs had lower coverage of high-quality mapped reads, as well as GC percentages in a very narrow range (Fig. 2). These contigs also tended to be smaller (<500 kbp). When we quantified nucleotide diversity in these using kmer content, we found that it was extremely low (Fig. 2a), with the majority having <50% of all 21-mers being unique (Methods). For this reason, we suspected that these contigs had been artificially collapsed during assembly, resulting in very few reads mapping with high quality. As expected, when we calculated depth based on all mapped reads rather than just high-quality mappers, these contigs exhibited very high coverage (Fig. 2b). Upon alignment to the *T. truncatus* genome, which is one of the most well-curated delphinoid assemblies, we found that 181 out of the 254 highly repetitive contigs (unique kmer content <90%) mapped to unplaced *T. tursiops* scaffolds, and were enriched for the telomeric motif TTAGGG (Zhong *et al*. 1992): 235 out of 254 were more than 4-fold enriched for this motif compared to only 16 of the 640 nonrepetitive contigs. This suggests that some of these are artifactually collapsed subtelomeric or telomeric regions.

To further characterize these problematic contigs, we examined the repeat element content. We first used RepeatMasker to determine repetitive genomic elements. 51% of the genome was designated as consisting of repetitive elements, being dominated by 111 Mbp of SINE elements (4.68% of the total genome), 704 Mbp of LINE elements (29.6%), and 236 Mbp of LTR elements (9.90%). We next used RepeatModeler to find de novo lineage-specific elements. This resulted in an additional 165.3 Mbp (6.93%) being designated as repetitive, consisting primarily of 30.9 Mbp of SINE elements, 115.6 Mbp of LINEs, 9.65 Mbp of LTR elements, and 2.25 Mbp of DNA transposons. Notably, the low nucleotide diversity contigs (few unique kmers) were masked only after this second step employing RepeatModeler, suggesting that they consisted almost exclusively of lineage-specific repetitive elements (Fig. 2c). This supports the hypothesis that these contigs are not collapsed due to repetitive elements; rather they are collapsed due to extremely low nucleotide diversity and are possibly telomeric, centromeric, or parts of the Y chromosome.

### Annotation

We annotated the repeat-masked genome with Braker3, resulting in 21,461 protein coding genes with an average length of 288 amino acids and a maximum length of 4,471 amino acids. These consisted of 119,057 exons and 97,627 introns with mean lengths of 156 and 1,605 bp, respectively. The total number of protein coding genes is larger than the number annotated in *T. truncatus* (19,240) despite the *T. truncatus* assembly having lower BUSCO scores. This is most likely due to differences in annotation methods, as *T. truncatus* was annotated using the NCBI eukaryotic genome annotation pipeline, which relies on Prosplign and Gnomon.

To check the accuracy and completeness of the final polished and purged nuclear assembly, we repeated the compleasm analysis. The final assembly exhibited slightly improved BUSCO scores in Laurasiatheria (98.37%) and averaged 98.3% single-copy complete BUSCOs across 5 different lineages (Supplementary Table 3 in File 1).

### Completeness of final assembly

**Fig. 3.**
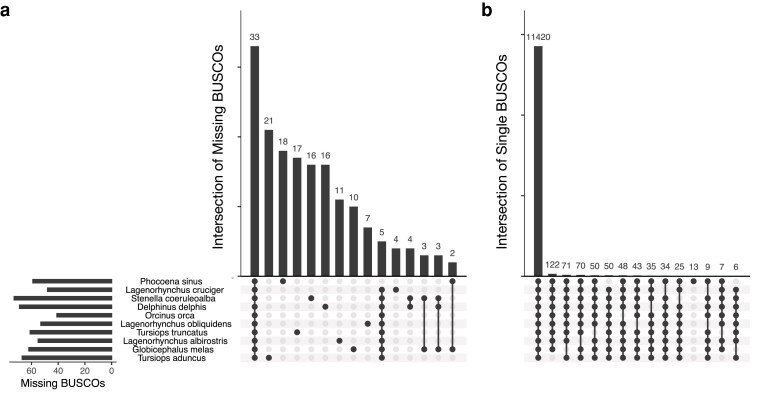
Upset plots of BUSCO overlaps across Delphinoidea assemblies. a) Intersection of single copy and complete BUSCOs using the Laurasiatheria ODB10 database. Each black point on a vertical line indicates taxa that share a set of BUSCOs, while each gray point indicates that the set of BUSCOs is missing from that taxon. The top bars show the total number of BUSCOs in each set (for example, 11,420 BUSCOs are present as single-copy complete in all 10 taxa; 122 are single-copy complete in all taxa except *T. aduncus*). The left bars indicate the total number of single-copy complete BUSCOs in each taxon. As in Table 1, the species are ordered by the number of total single-copy complete BUSCOs. b) Intersection of missing BUSCOs. As in (a), overlaps in BUSCO sets are indicated by black points and taxa that are not part of that set are in gray. Thirty-three BUSCOs are missing from all Delphinoidea, most of which were likely lost in the ancestor of these taxa. For both plots, only the 15 intersections with the most BUSCOs are shown, as there are a large number of intersections that contain only 1 or 2 BUSCOs.

We compared the completeness of the hourglass assembly to 9 other Delphinoidea assemblies, which we selected on the basis of their being the most contiguous and complete assemblies in this superfamily (all are designated as reference genomes by NCBI). However, we also excluded several genomes designated as reference quality as they had far fewer single-copy complete BUSCOs, including *Steno bredanensis*, *Grampus griseus*, and *Sousa chinensis*. We found that the hourglass assembly had more complete and single-copy BUSCOs (12,056; Table 2) than any other assembly except the vaquita, *Phocoena sinus* (12,072); correspondingly, it had fewer missing BUSCOs (48 missing) than any other assembly except the orca, *Orcinus orca* (41 missing; Fig. 3). 33 of these BUSCOs were missing from all Delphinoidea assemblies, and were therefore likely lost in the ancestral lineage. If these were indeed lost, this suggests the quality of the hourglass assembly is even higher than first appears: excluding the 33 BUSCOs missing in all, while the hourglass is missing only 15 out of 12,201, the average reference-level Delphinoidea assembly is missing 26.

**Table 2.** Assembly completeness across Delphinoidea.

Species	Single copy	Duplicated	Missing	Fragmented
*Phocoena sinus*	12,072 (98.68%)	80 (0.65%)	59 (0.48%)	23 (0.19%)
*Lagenorhynchus cruciger*	**12,056 (98.55%)**	**96** (**0.78%)**	**48** (**0.39%)**	**34** (**0.28%)**
*Stenella coeruleoalba*	12,055 (98.54%)	87 (0.71%)	73 (0.60%)	19 (0.16%)
*Delphinus delphis*	12,049 (98.49%)	94 (0.77%)	69 (0.56%)	22 (0.18%)
*Orcinus orca*	12,048 (98.48%)	129 (1.05%)	41 (0.34%)	16 (0.13%)
*Lagenorhynchus obliquidens*	12,043 (98.44%)	112 (0.92%)	53 (0.43%)	26 (0.21%)
*Tursiops truncatus*	12,028 (98.32%)	119 (0.97%)	61 (0.50%)	26 (0.21%)
*Lagenorhynchus albirostris*	12,013 (98.19%)	135 (1.10%)	55 (0.45%)	31 (0.25%)
*Globicephala melas*	12,002 (98.10%)	148 (1.21%)	62 (0.51%)	22 (0.18%)
*Tursiops aduncus*	11,969 (97.83%)	157 (1.28%)	67 (0.55%)	41 (0.34%)

Each column indicates the total number (percentage) of single-copy complete, duplicated, missing, or fragmented BUSCOs as found by Compleasm. The species are ordered by the number of total single copy complete BUSCOs. The statistics for *L. cruciger* are bolded.

The number of duplicated BUSCOs in the hourglass was 20% lower than the average across the selected Delphinoidea comparison species (96 vs 116); however, the number of fragmented BUSCOs was 30% higher than the average (34 vs 26). This may be an indication that Oxford Nanopore data results in an increased number of uncorrected small insertions and deletions resulting in truncated reading frames compared to the combination of PacBio and Illumina data used for most of the other assemblies (Supplementary Table 1 in File 1).

We found that the BUSCO results correlated with patterns expected from the evolutionary relationships of the taxa. 50 BUSCOs were present as single copy and complete in all taxa except the most diverged taxon, the vaquita; similarly, the vaquita contained 13 single copy and complete BUSCOs that were not single copy and complete in all other taxa (Fig. 3). Nine single-copy complete BUSCOs were not single-copy complete in both the hourglass and *Lagenorhynchus obliquidens*, the Pacific white-sided dolphin, but were present as single-copy complete in all others. This supports the proposal that the hourglass and Pacific white-sided dolphin are likely sister species to the exclusion of *L. albirostris*, although additional phylogenetic analyses are needed to confirm this. Similar patterns were apparent for both *Tursiops* sister species.

Finally, we found that the annotated mitochondrial genome had the full complement of 22 tRNAs, 12S and 16S ribosomal RNAs, and 13 coding sequences expected for a vertebrate mitogenome.

### Variant calling and phasing

We called heterozygous sites using both Clair3 and DeepVariant. These have been shown to be the most accurate Oxford Nanopore variant callers, with median indel F1 scores above 99.5% and SNP F1 scores at or above 99.99% at 50× depth in bacterial genomes (Hall *et al*. 2024).

Clair3 called 4,848,349 million SNVs, 620,899 insertions, and 516,291 deletions. When we filtered these to qualities above 14, this resulted in 4,223,575 SNVs, 208,561 insertions and 278,677 deletions. DeepVariant called 6,869,645 SNVs, 1,069,178 insertions and 3,381,036 deletions, which when filtered to those with quality 20 or more, resulted in 4,188,921 SNVs, 327,370 insertions, and 287,574 deletions. Overlapping the 2 call sets resulted in a total of 4,021,582 high-quality SNPs, 181,409 insertions, and 246,147 deletions (note that the designation of calls as insertions or deletions arbitrarily depends on the state of these positions in the haploid genome assembly).

We also examined the distribution of polymorphic sites across the genome. We hypothesized that due to selection, exons should harbor fewer SNPs relative to introns and intergenic regions, and that exons should harbor few indels. Of the high confidence SNP calls, 0.551% was within exons. This contrasts with the 0.853% of the genome that is annotated as exonic. 6.83% SNPs calls were within introns, closely matching the 6.60% of the genome that is intronic. For indels, only 0.245% lay within exons, a 3.5-fold depletion compared to genome-wide. In addition, exons were depleted for indels that were not multiples of 3 bp in length; this was not true for introns (Supplementary Fig. 4 in File 1).

At first glance, there appears to be little depletion for 1 bp indels in exons (Supplementary Fig. 4 in File 1). This is possibly due to greedy annotation of some exonic regions that are in fact pseudogenes. To test this, we looked at the precise distribution of indels across genic regions. In total, we found 428 1 bp indels in exons spread across 346 coding protein coding genes (1.6% of all protein coding genes; Supplementary Fig. 5 in File 1), compared to 17,013 1 bp indels in introns across 6,270 genes (37% of all protein coding genes). Exonic indels were highly concentrated, with 140 contained in only 58 genes. This was not due simply to longer genes harboring more indels: on a per kbp basis, shorter genes had higher indel rates (Supplementary Fig. 5 in File 1). The higher indel rate in short genes indeed suggests that some may be pseudogenes or not coding, and thus less constrained in terms of selection on indels. Overall, we found that on a per kbp basis exons harbored ∼10-fold fewer indels than introns (Supplementary Fig. 5c and d in File 1). This analysis suggested that the majority of exonic 1 bp indels are not in fact due to misassembly or mistakes in error-correction, but instead lack of selective constraint. Nevertheless, some may be due to assembly errors. These are most likely to occur at homopolymeric runs. It is also possible that there is an excess of homopolymers in introns relative to exons, and this accounts for the higher indel rates we observe; additional analyses of the locations, types, and functional effects of these indels would be required to yield insight into this.

**Fig. 4.**
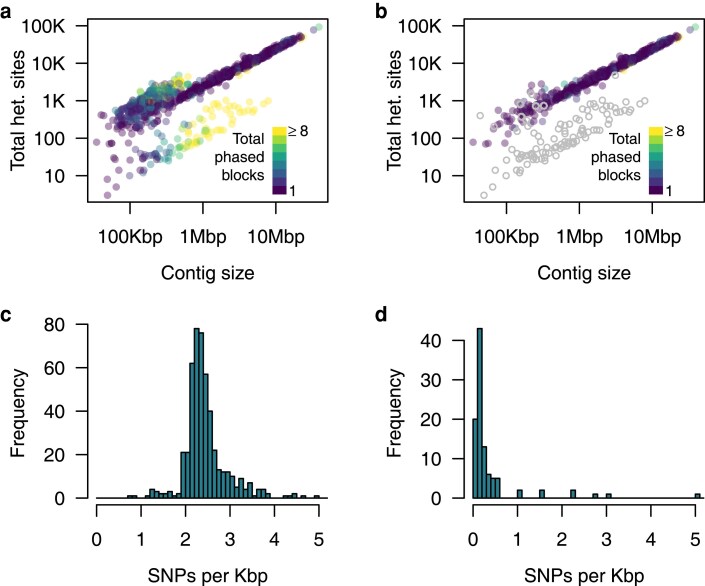
The majority of contigs are phased into a single block. a) Number of phase blocks per contig relative to contig size and the number of heterozygous sites in each contig. The majority of contigs that are not fully phased into a single block have low levels of polymorphism (green and yellow points) except for a small number of very large contigs that could not be fully phased (upper left points above the diagonal). b) The same plot as in panel (a) but with repetitive contigs removed (defined here as contigs with <90% unique 21-mers and putative sex chromosome contigs (coverage <30) colored in gray. The sex chromosome contigs are inferred as having ∼20-fold lower levels of polymorphism, likely resulting from a combination of assembly errors, false positive calls, and the homologous pseudoautosomal regions in the X and Y chromosomes. c) The number of SNPs per kbp in nonrepetitive contigs (>90% unique 21-mers) and with >45-fold coverage of high-quality mappers (presumed autosomal contigs). Most autosomal contigs have ∼2.4 heterozygous SNPs per kbp with very little deviation from this average. d) The number of SNPs in non-repetitive contigs with <30-fold depth (presumed sex chromosome contigs). These contigs averaged 0.11 SNPs per kbp.

Finally, we used Whatshap (Shafin *et al*. 2021) to phase the genome. 55% of all contigs were phased into a single block, and 80% were phased into 3 or fewer blocks (Fig. 4; Supplementary Fig. 6). However, without additional information, it is difficult to say whether there was a substantial amount of haplotype switching in these phased blocks, especially in regions of low polymorphism.

The genome assembly we present here is one of the most complete of any cetacean, with BUSCO completeness above 98%. Despite relying solely only on Oxford Nanopore data, this quality exceeds many recent cetacean assemblies, even those relying on multiple sequence technologies (e.g. PacBio and Illumina; Table 2; Supplementary Table 1; Morin *et al*. 2020). Overall, the results here illustrate the potential for inexpensive, and more importantly, accessible, assembly of large genomes, enabling community participation and consideration of Indigenous aspirations, particularly data sovereignty (Garrison *et al*. 2019). This high-quality genome should assist considerably in resolving taxonomic uncertainties in the subfamily Delphininae. In addition, by providing a comprehensive genomic resource, this study will contribute to a deeper understanding of cetacean evolution and facilitate informed conservation efforts for this enigmatic species.

## Supplementary Material

jkaf044_Supplementary_Data

## Data Availability

As noted, this species and data are considered taonga by Ōraka-Aparima. Therefore, the basecalled sequence data, assembly, annotations, and vcf files have been deposited at the Aotearoa Genomic Database Repository (https://data.agdr.org.nz/, dataset ID AGDR00046), a data repository that follows both the FAIR (findable, accessible, interoperable and reusable) and CARE (collective benefit, authority to control, responsibility and ethics) principles. The data will be made available for scientific and conservation-related purposes on behalf of Ōraka-Aparima Rūnaka, with requests for commercial applications deferred to them. A github repository outlining the steps of the assembly and the code and data necessary for plotting the figures presented here is available (https://github.com/osilander/hourglass-assembly). Supplemental material available at G3 online.
