# Supplementary material for: A high-quality Oxford Nanopore assembly of the hourglass dolphin (Lagenorhynchus cruciger) genome
Source: G3 (Bethesda). 2025 Feb 28;15(5):jkaf044. doi: 10.1093/g3journal/jkaf044 (PMC12060246; doi:10.1093/g3journal/jkaf044)
Supplement: jkaf044_Supplementary_Data [file jkaf044_supplementary_data.pdf]

Table S1. List of other Delphinoidea genomes.

Organism	Assembly ID	Date	Contigs	Technology
Tursiops aduncus	GCA_003227395.1	Jun 14, 2018	44,281	Illumina HiSeq; Illumina NovaSeq
Lagenorhynchus obliquidens	GCA_003676395.1	Oct 23, 2018	21,792	Illumina HiSeq; Illumina NovaSeq
Phocoena sinus	GCA_008692025.1	Sep 26, 2019	272	PacBio Sequel I; Illumina NovaSeq; Arima Genomics Hi-C; Bionano Genomics DLE-1
Tursiops truncatus	GCA_011762595.1	Mar 27, 2020	1,035	PacBio Sequel I CLR; Illumina NovaSeq; Arima Genomics Hi-C; Bionano Genomics DLS
Orcinus orca	GCA_937001465.1	May 3, 2022	570	PacBio and Hi-C
Lagenorhynchus albirostris	GCA_949774975.1	Apr 8, 2023	1,479	PacBio and Arima2 Hi-C
Delphinus delphis	GCA_949987515.1	May 1, 2023	1,598	PacBio and Arima2 Hi-C
Stenella coeruleoalba	GCA_951394435.1	Jun 16, 2023	1,630	PacBio and Arima2 Hi-C
Globicephala melas	GCA_963455315.1	Sep 21, 2023	2,076	PacBio and Arima2 Hi-C

Table S2. BUSCO completeness in Laurasiatheria across basecallers and assemblers.

Assembler	Basecaller	Single copy	Duplicate	Fragmented	Missing
Goldrush	Guppy	11,795 (96.4%)	210 (1.72%)	98 (0.80%)	131 (1.07%)
Goldrush	Dorado	11,826 (96.7%)	191 (1.56%)	92 (0.75%)	124 (1.01%)
Nextdenovo	Guppy	11,937 (97.6%)	118 (0.96%)	82 (0.67%)	96 (0.78%)
Nextdenovo	Dorado	11,936 (97.6%)	116 (0.95%)	78 (0.64%)	104 (0.85%)
Raven	Guppy	12,017 (98.2%)	109 (0.89%)	58 (0.47%)	50 (0.41%)
Raven	Dorado	12,024 (98.3%)	107 (0.87%)	55 (0.45%)	48 (0.39%)
Raven	Dorado Q16	12,016 (98.2%)	104 (0.85%)	58 (0.47%)	56 (0.46%)

Table S3. BUSCO completeness of the polished Dorado-basecalled Raven hourglass assembly relative to four mammalian lineages and to all eukaryotes.

Lineage	Single copy and complete	Duplicated	Fragmented	Missing
Laurasiatheria	12,056 (98.55%)	96 (0.78%)	34 (0.28%)	48 (0.39%)
Cetartiodactyla	13,118 (98.37%)	118 (0.88%)	67 (0.50%)	32 (0.24%)
Eutheria	11,171 (98.29%)	75 (0.66%)	49 (0.43%)	70 (0.62%)
Mammalia	9,088 (98.50%)	63 (0.68%)	34 (0.37%)	41 (0.45%)
Eukarya	250 (98.04%)	5 (1.96%)	0 (0%)	0 (0%)

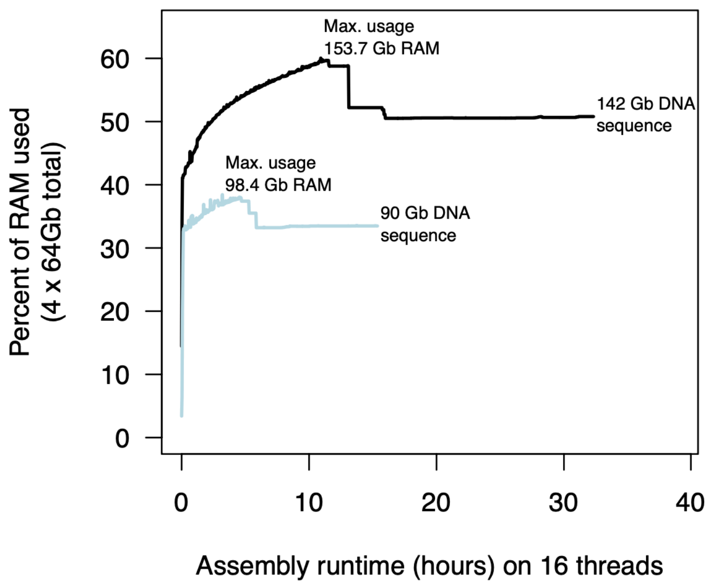

Figure S1. RAM usage during Raven genome assembly. The plot shows the amount of RAM used by Raven when performing assembly with 16 threads and using sequencing datasets with either 142 Gbp (the full dataset presented here) or 90 Gbp (a subset filtered by prioritising longer reads). The assembly from the 142 Gbp dataset required a maximum of 153.7 Gb of RAM, beyond the scale of most consumer laptops. The initial assembly from 142 Gbp data had 1,243 contigs, 2.44 Gbp total size, and an N50 of 8.08 Mbp. The initial assembly from the 90 Gbp dataset was slightly lower quality (1,621 contigs, 2.47 Gbp total length, N50 5.85 Mbp), but required less than 100 Gb total RAM. Current high end laptops have up to 128 Gb of RAM (for example, the HP ZBook Fury 16 G10, configurable with 128 Gb RAM and an A1000 GPU for less than $3,000 USD).

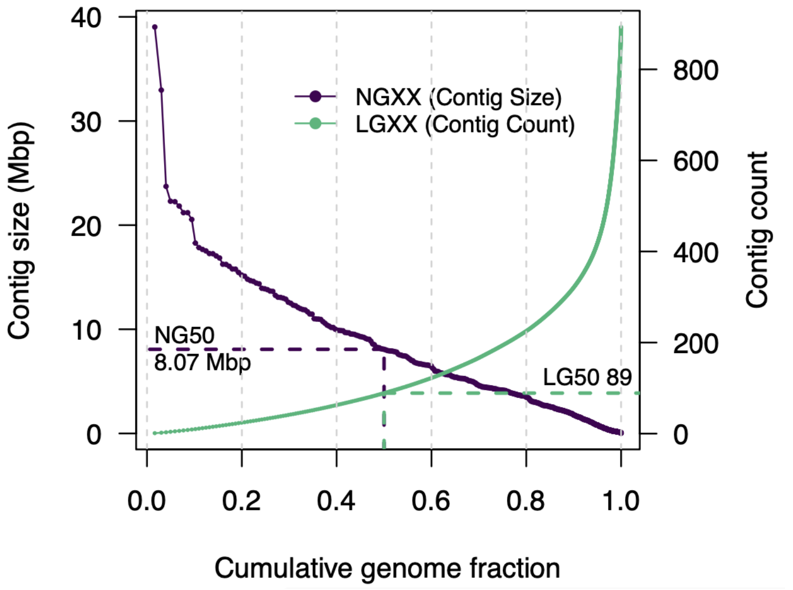

Figure S2. NG and LG statistics across the final haplotig purged and polished Raven assembly. The NG50 and LG50 values are indicated in purple and green, respectively.

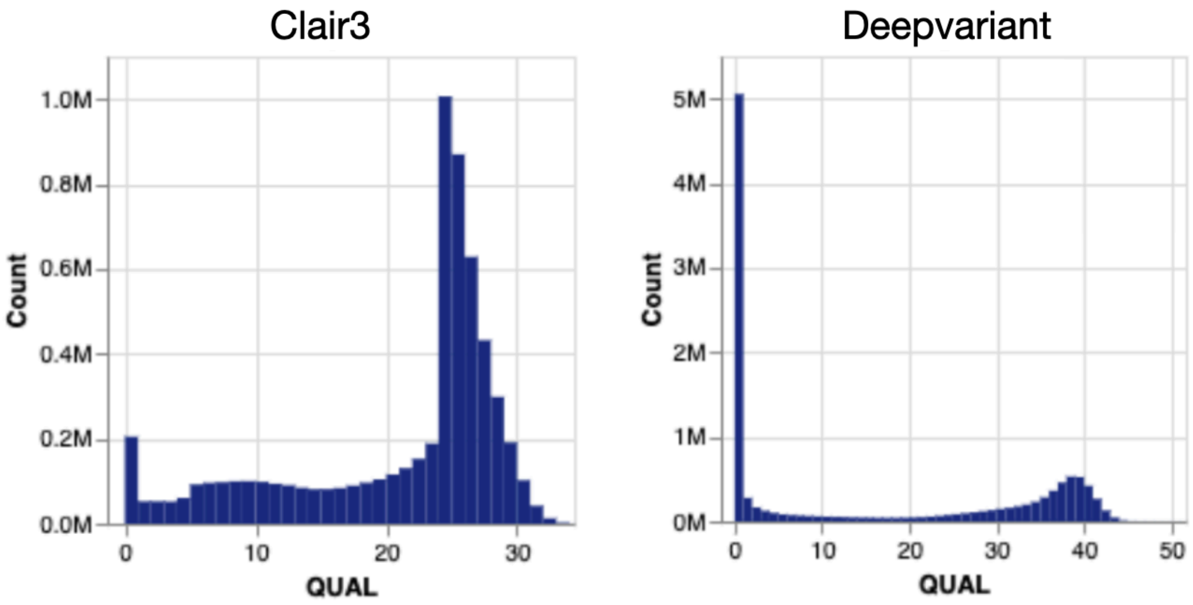

Figure S3. Genotype quality scores for Clair3 and DeepVariant. Clair3 calls have a local count minima adjacent to the high quality peak at around q14; Deepvariant at around q20. We filtered both callsets to include only variants with higher qualities than these, respectively, and then merged the two filtered callsets to form a set of conservative high quality genotype calls.

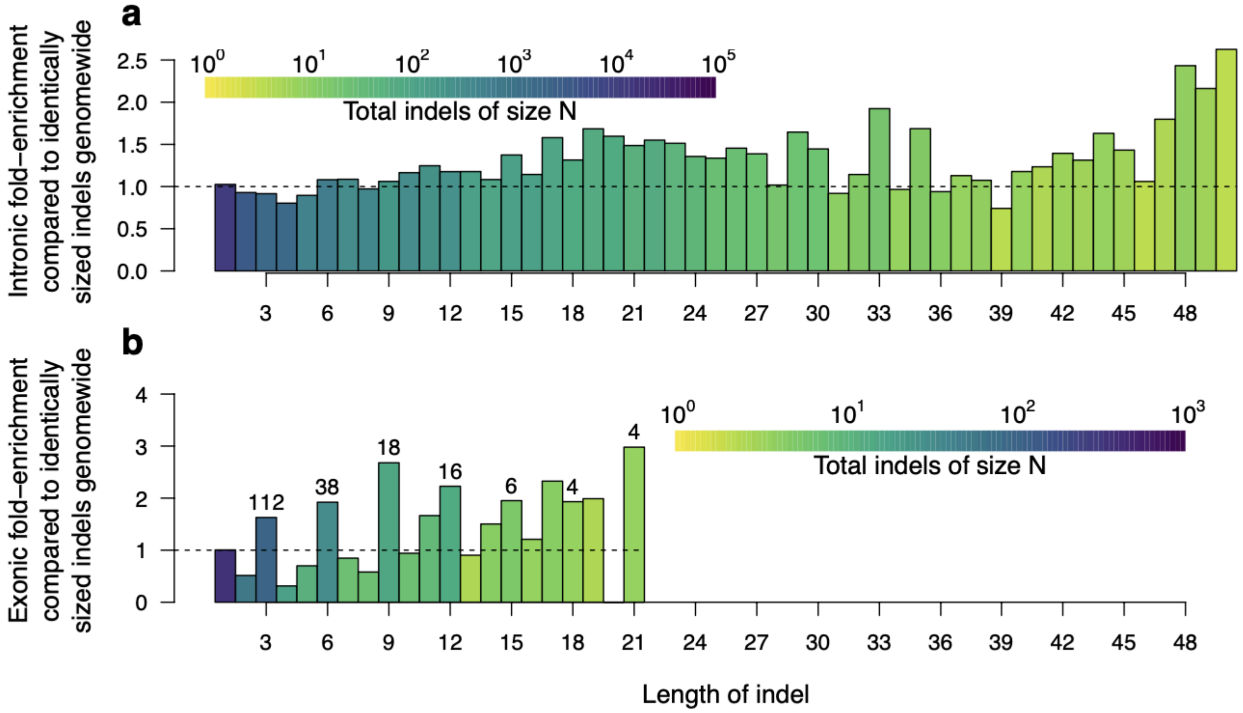

Figure S4. Enrichment and depletion of specific indel sizes in introns and exons relative to all genomic locations. A. The ratio of the number of indels of each size in introns relative to the number of indels of that size genome-wide. If there is no depletion or enrichment, the expected ratio is one (dotted line). Instead, there is a slight depletion of small intronic indels (two to five bp) and an enrichment of larger intronic indels (between five and 25 bp). Overall, we found a slight enrichment of indels in introns compared to the remainder of the genome (7.21% observed vs. 6.60% expected). **B**. The analogous plot as in (A) but for exonic regions. Here there is a specific depletion of indels that are not multiples of three bp. This is expected, as indels that are not multiples of three change the reading frame. For both panels, the color of the bar indicates the total number of indels at that size (on a log scale). For panel (B), above each bar that is a multiple of three, we show the total number of indels that are of that size. For example, we observed a total of 150 three bp indels in exons. Relative to the total genome-wide number of three bp deletions and normalising for the fraction of indels in exonic regions, we would have expected approximately 1/2 that number (dotted line). The total fraction of indels across all sizes in exonic and intronic regions must sum to one; thus, if some indel sizes are depleted, others must necessarily be enriched. We do not show the relative depletion or enrichment for exonic indels beyond 21 bp as these are small in number and fold-enrichment or depletion varies widely. Note that both the y-axes scales and colour scales are different on each plot.

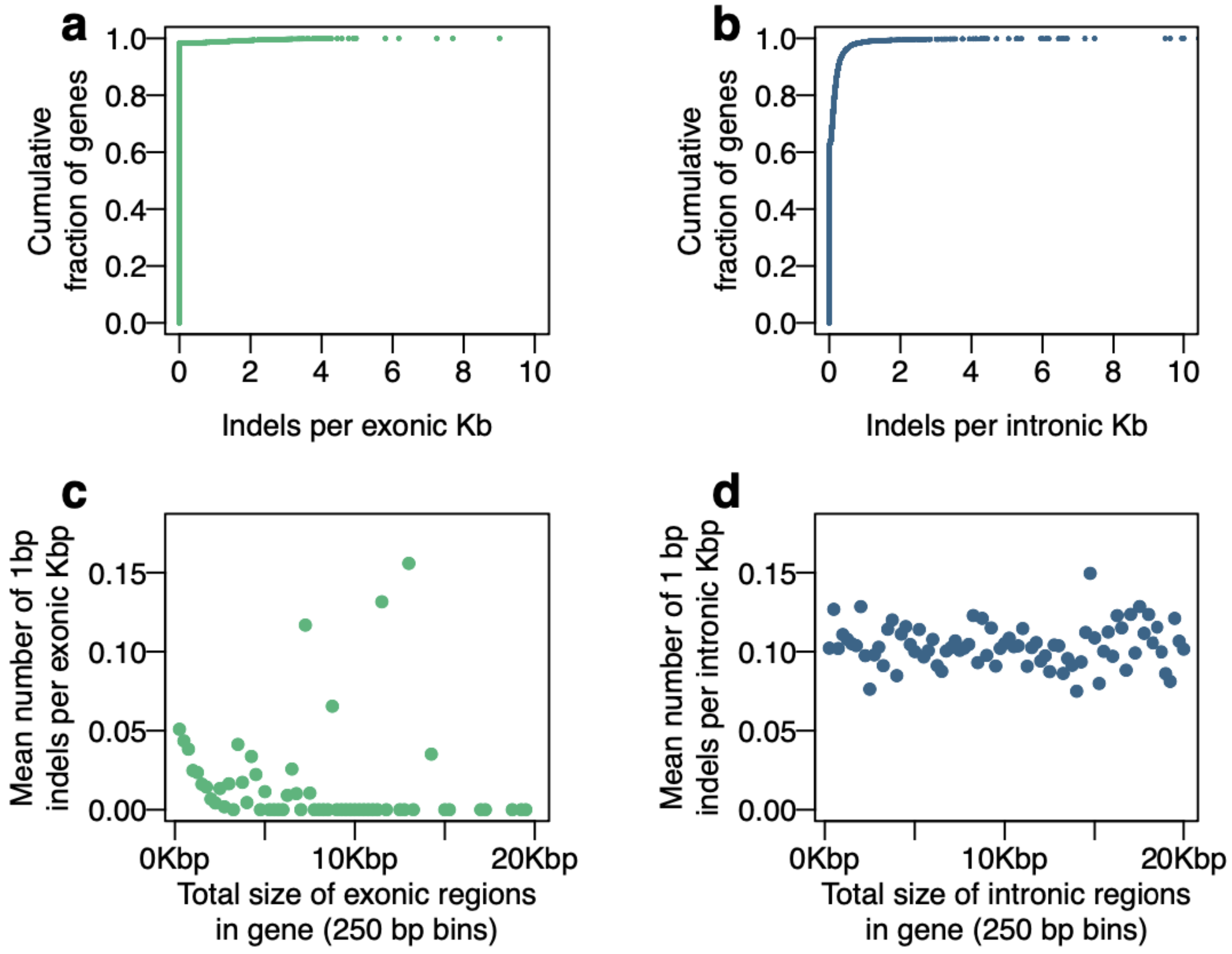

Figure S5. Substantial depletion of 1 bp indels in exonic regions A. Cumulative fraction of genes harboring exonic indels. The vast majority of genes (98.4%) have no indels. **B**. Cumulative fraction of genes harboring intronic indels. Almost 40% of all genes have intronic indels. However, intronic regions are much larger than exonic regions in most genes. **C**. Per kbp indel rates in exons. We added up the total length of exonic regions in each gene, and divided the genes into 250 bp bins according to their total exonic length. For all genes within a 250bp bin, we calculated the mean number of indels per kbp of exon. There is a slight enrichment of indels in shorter genes, suggesting that some may be pseudogenes, generally subject to lower levels of evolutionary constraint, or possibly increased rates of assembly error. There are also a small number of bins at longer lengths that have high mean indel numbers. In almost all cases, these are dominated by a single gene with a very high number of indels. For example, in the 7,000 to 7,250 bp bin, one gene has nine 1 bp indels (1.3 indels per kbp). The other ten genes in this bin have none; in the 8,500 to 8,750 bp bin, there is one gene with four 1 bp indels; the other six have none; in the 11,250 to 11,500 bp bin, one gene has six indels; the other three have none. **D**. Per kbp indel rates in introns. As for exons, we added up all intronic regions in a gene, divided the gene-intronic regions into 250 bp bins, and calculated the mean number of indels per kbp of intron within each bin. Over any intronic length, there are approximately 0.11 indels per kbp, approximately 10-fold higher than for exons.

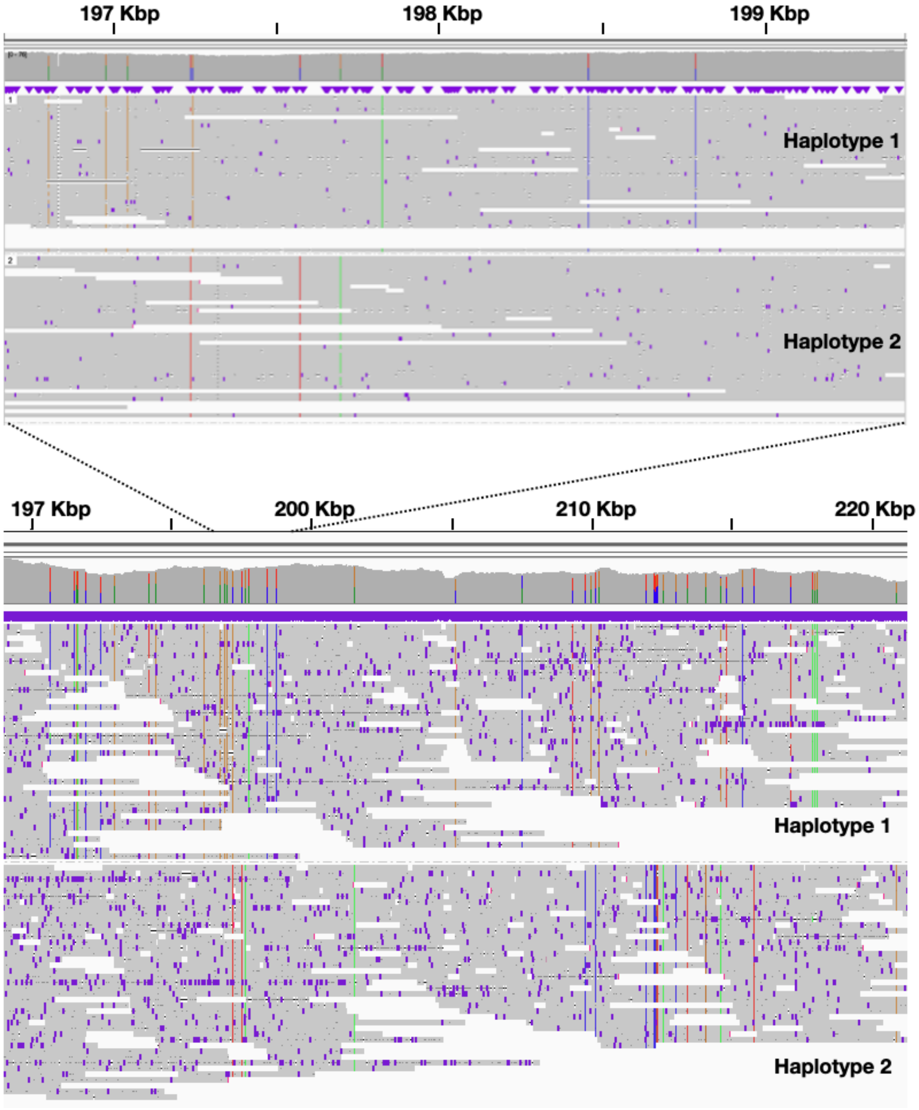

Figure S6. Phased haplotigs at 1 kbp (top) and 10 kbp (bottom) scales. Long Oxford Nanopore 10.4.1 chemistry reads allow phasing across the genome, despite relatively low levels of polymorphism in some regions (e.g. fewer than ten SNPs in the 10 kbp region between 200 kbp and 210 kbp above).
